# Risk levels for suffering a traffic injury in primary health care. The LESIONAT* project

**DOI:** 10.1186/1471-2458-10-136

**Published:** 2010-03-16

**Authors:** Carlos Martín-Cantera, Daniel Prieto-Alhambra, Lydia Roig, Susana Valiente, Katherine Perez, Luis Garcia-Ortiz, Jordi Bel, Fernando Marques, Xavier Mundet, Xavier Bonafont, Marti Birules, Núria Soldevila, Elena Briones, LESIONAT Research Group

**Affiliations:** 1 Departament of Medicine, Universitat Autònoma de Barcelona, Barcelona, Spain; 2 ICS, ABS Passeig Sant Joan, SAP Dreta Barcelona, Unitat de Suport a la Recerca Barcelona Ciutat, Barcelona, Spain; 3 IDIAP Jordi Gol, Grupo Investigación Cardiocat, Preventive Services and Health Promotion Research Network -redIAPP, Barcelona, Spain; 4 ICS, ABS Passeig Sant Joan, SAP Dreta Barcelona, Departament of Medicine, Universitat Autònoma de Barcelona, IDIAP Jordi Gol, Barcelona, Spain; 5 ICS, ABS La Garriga, La Garriga, Spain; 6 ICS, ABS Ramon Turro, Barcelona, Spain; 7 Agencia de Salut Pública de Barcelona, Barcelona, Spain; 8 Primary Care Research Unit, La Alamedilla Health Centre, Preventive Services and Health Promotion Research Network -redIAPP, Salamanca, Spain; 9 ICS, ABS Via Barcino, Barcelona, Spain; 10 ICS, ABS Cervera, Lleida, Spain; 11 Departament of Medicine, Universidad Autónoma de Barcelona ABS Carmel, Preventive Services and Health Promotion Research Network -redIAPP, Barcelona, Spain; 12 Servicio de Farmacia Hospitalaria, Hospital Germans Trials Pujol, Badalona, Spain; 13 ICS, ABS Poblenou, Barcelona, Spain; 14 ICS, ABS La Mina, Barcelona, Spain; 15 Grupo Investigacion Cardiocat. Preventive Services and Health Promotion Research Network -redIAPP, Barcelona, Spain

## Abstract

**Background:**

Literature shows that not only are traffic injuries due to accidents, but that there is also a correlation between different chronic conditions, the consumption of certain types of drugs, the intake of psychoactive substances and the self perception of risk (Health Belief Model) and the impact/incidence of traffic accidents. There are few studies on these aspects in primary health care.

The **objectives **of our study are:

Main aim: To outline the distribution of risk factors associated with Road Traffic Injuries (RTI) in a driving population assigned to a group of primary health care centres in Barcelona province.

Secondly, we aim to study the distribution of diverse risk factors related to the possibility of suffering an RTI according to age, sex and population groups, to assess the relationship between these same risk factors and self risk perception for suffering an RTI, and to outline the association between the number of risk factors and the history of reported collisions.

**Methods/Design:**

Design: Cross-sectional, multicentre study.

Setting: 25 urban health care centres.

Study population: Randomly selected sample of Spanish/Catalan speakers age 16 or above with a medical register in any of the 25 participating primary health care centres. N = 1540.

Unit of study: Basic unit of care, consisting of a general practitioner and a nurse, both of whom caring for the same population (1,500 to 2,000 people per unit).

Instruments of measurement: Data collection will be performed using a survey carried out by health professionals, who will use the clinical registers and the information reported by the patient during the visit to collect the baseline data: illnesses, medication intake, alcohol and psychoactive consumption, and self perception of risk.

**Discussion:**

We expect to obtain a risk profile of the subjects in relation to RTI in the primary health care field, and to create a group for a prospective follow-up.

**Trial Registration:**

Clinical Trials.gov Identifier: NCT00778440.

## Background

Traffic injuries are serious health problems that cause death, morbidity and disability, as well as an important impact on a country's economy [1]. There are three high risk groups: 15-24 years old who use mopeds or motorbikes, 18-34 years old driving as tourists, and over 65 years old pedestrians in urban areas. According to the last Catalan Health Survey (Enquesta de Salut a Catalunya ESCA) in 2006, 17.8% of those over 15 years of age had been injured in a road traffic accident (RTI) in the last year, 6.75% of whom had been injured in a road traffic collision in the previous two weeks. Figures for men were higher at 9.33% than for women at 4.33%. There were also differences in age with 8.05% aged between 15 and 44, 5.04% aged between 45 and 64 and 15.5% aged over 65 [2].

RTI are not coincidental, but they adhere to foreseeable factors. Medication and chronic conditions, despite not being the main implicated factors, are related to some cases [3-5]. Usually, when we talk about implicated factors in RTI, four groups can be considered: human factors, aspects related to the vehicle, aspects related to the collision environment (traffic, lane, etc) and socio-economic aspects [6,7].

The alcohol consumption factor on its own is the biggest contributor to the production of serious RTIs. The use of certain types of medication and psychoactive substances may alter the driving capacity as well [8]. In our field, between 5% and 15% of drivers killed in an accident show significant blood levels of other psychoactive substances (including or not including alcohol) [9]. There have also been international studies which have proven the existence of a relationship between psychoactive substances like benzodiazepines [10,11], cannabis and cocaine [12,13].

As for health problems, the previous morbidity of the driver is considered to be a possible accident inducing factor, and some chronic health problems like diabetes mellitus, epilepsy, hearing or visual impairments, cognitive deteriorations and some psychiatric disorders have been associated with RTI. Despite this, the illness-accident relationship is not easy to consider objectively due to methodological difficulties [14].

### Drugs and psychoactive substances

According to the 2006 Catalan Health Survey (ESCA'06), 21.7% of the Catalan population take some kind of medication, 69.5% of which is prescribed by a doctor, 5.5% prescribed by a pharmacist and 25% is self-medicated. Table 1 shows the available data on the consumption of certain medication which can affect driving capacities in the general population of Catalonia [2].

**Table 1:** Consumption of certain drugs in the general population of Catalonia.

Pharmacological Group	General population (%)	Men (%)	Women (%)
Anxyolitics	9,1	5,72	12,38
Antidepressants	6,8	3,73	9,77
Antihistamines	3,18	2,44	3,89
Hypnotics	7,32	4,02	10,62
Antiepileptic drugs	0,3	0,28	0,30
Antiparkinsonian agents (>65 yrs)	0,76	0,6	0,8

ESCA'06

In a study carried out on the Spanish driving population killed in a traffic accident in 1992, 45% had been taking some kind of medication during the previous year, 17% of which were taken regularly [15].

On the other hand, a study which included 8,000 Spanish drivers showed that an estimated 10% of those killed or injured in a traffic collision had taken some kind of psychoactive substance. Moreover, an analysis by the National Toxicology Institute (Instituto Nacional de Toxicología) during the period between 1991 and 1999 showed that in 4.382 drivers killed on the road, 54% had taken some kind of psychoactive substance and 5% had been on medication [16,17]. Finally, a recent study, performed in Catalonia, which assessed subjects who had been injured in a traffic accident, observed a high prevalence of consumption of psychoactive substances, especially alcohol, cannabis and cocaine, which was relevant in all age groups [18].

Several review papers underline the effect of certain drugs on driving capacity such as narcoleptics or antipsychotics, anxyolitics, sedatives and hypnotics, tricycle antidepressants, lithium, (narcotic and non narcotic) analgesics and anti-migraine agents, anaesthetics, antiepileptic drugs, non steroid anti-inflammatory drugs, mio-relaxing agents, anti-Parkinson drugs, H1-antihistamines, anticolinergics and some cardiovascular drugs [19,20].

A recently published review paper suggests the need for research on the role of medication in road safety (other than the use of benzodiazepines, which has already been widely studied) [21], and the need to investigate more on the side effects of different drugs when driving [22].

In Spain, the cautionary information about the effect of drugs on driving capacities is ruled by Royal Law (Real Decreto) 1345/2007 [23], which newly includes a pictogram for any medication that can alter driving ability or the ability to use dangerous machinery. This information is currently being reviewed and classified according to the ICADTS (International Council on Alcohol, Drugs and Traffic Safety) categories, shown in Table 2. These take into consideration 3 groups according to the alcohol intake amounts considered safe, risky or not recommended. Currently, 424 active principles have been reviewed, which will lead to a change in 2,633 medicine labels by 2011, as in other eleven EU countries [24].

**Table 2:** ICADTS classification of drugs in terms of driving effects

CATEGORY	DESCRIPTION	BAC (g/l)	ADVISE TO THE PATIENT
I	Presumed safe or unlikely effect	< 0,5	Read the patient information leaflet before driving.
II	Likely minor or moderate effect	0,5 - 0,8	Do not drive without consulting a health professional.
III	Likely severe effect or presumed dangerous	> 0,8	Do not drive while you are taking this medicine.

BAC: Blood Alcohol Concentration (g/l) equivalence.

The European Union has also dealt with problems related to medication and driving, particularly in an international project named "The Integrated Project DRUID (Driving Under the Influence of Drugs, Alcohol and Medicines)", which analyses the effect that the intake of medicines, medicinal herbs and psychoactive substances can have on road safety [25].

### Chronic health conditions

Until the 1960's, the correlation between chronic health problems and road traffic injuries was very controversial. Different groups of researchers started to observe a correlation between collisions and injuries suffered by drivers with diabetes, epilepsy, cardiovascular illnesses, alcoholism and mental disorders [26].

Some ongoing studies are currently studying the effect that diverse long-term conditions and age, elements that usually go together, can have on the incidence of RTIs [27].

The Catalan Accident Statistics Annual Report (2003) indicates that in 7.6% of the collisions with victims, drivers suffered from the sudden appearance of an illness [28]. This same observation has been suggested at an international level [29]. According to data obtained from diverse review papers [30-33], a risk table has been published for different health problems and their adjusted risk, if the correct treatment was carried out. The following findings should be noted: the relative risk of a driver with a chronic health condition of suffering a traffic injury is 1.33 (IC95% 1.28-1.37); the most highly associated illnesses are: Sleep Apnea Syndrome [RR 3.71 (IC95% 2.14-6.4)], neurological conditions [RR 1.75 (IC95% 1.61-1.89)], arthritis/joint limitations [RR 1.17 (IC95% 1.004-1.36)], cardio-vascular illnesses [RR 1.23 (IC95% 1.09-1.38)], diabetes mellitus [RR 1.56 (IC95% 1.31-1.86)], mental disorders [RR 1.72 (IC95% 1.48-1.99)], visual impairments [RR 1.09 (IC95% 1.04-1.15)], hearing impairments [RR 1.19 (IC95% 1.02-1.4)] and high alcohol consumption [RR 2.00 (IC95% 1.89-2.12)] [30].; and the risks for the population with chronic health problems diminish in general if they are being treated and adequately controlled, with the exception of some chronic health problems where the risk can increase through the use of different types of medication (e.g. benzodiazepines) [31,34].

### The situation in Primary Care

In our country, few articles have been published on RTI and their impact in Primary Health care. Most of them, written during the 1990's have consisted of descriptive epidemiological studies on the impact of road traffic injuries on the number of emergency consultations, its prevalence according to age groups and gender and studies on time, place and gravity of the RTI. If summarised, they show that the frequency of RTI is approximately 8 per 1,000 inhabitants [35,36] making up 4.1% of all consultations in a primary health care centre [37]. Of all collision accidents of any kind, those on a public road are the second most frequent, after domestic accidents, road traffic injuries making up between 8.3% and 8.6% of all kinds [37-39].

The most serious RTI happen on the road, and affect male drivers more (approximately 70%), with an average age of 27 ± 3 years [35-37], with a large peak in the group around 20 ± 5 years of age (42.8% of cases) [36]. The impact makes up 1% of the total of patients attended in an emergency centre [40].

Diverse authors warn of the high occurrence of RTI in children (245 per 1,000 inhabitants) [38], and the importance of the use of child protection methods in vehicles. 20.75% of child accidents correspond to the RTI that affect those aged 12 ± 2 years [41,42].

### The Theory of Change model and risk of RTI

There are different theoretical explanations which try to predict the change in the prevention behaviour of populations. Of all the analysed theories [43], the so called Health Belief Model shows that the most important elements are: risk perceptions (personal susceptibility to suffer an injury), capacity to carry out effective activities and their economic costs.

Several studies have aimed at describing the risk perception in relation to risky driving behaviours [44], cultural differences [45], the use of mobile phones while driving [46], the position of hands when driving [47]. Diverse population groups have also been studied such as young drivers compared to older drivers [48]. Others have examined differences in the perception of traffic risks for older and younger adults [49]. Also, the perception of risk has been studied in relation to training [50], age and driving expertise [51]. These have been particularly assessed amongst drivers under the effects of alcohol [52,53]. Just a few studies have established the existing relationship between health conditions and risk perception; amongst them, we found some papers on risk perception in diabetic patients during hypoglycaemic stages [54,55], and in epileptic subjects [56].

Available publications show several methods to measure the risk perception for an RTI. Likert scales with closed questions and a scale between complete disagreement to complete agreement have been used in some cases [47], as well as multiple-choice questionnaires (measuring probability of traffic accidents, consequences of traffic accidents, risk sensitivity and risk willingness with Likert scales) [45]. In some studies, the risk associated to particular traffic conditions and driving behaviours was assessed [50] and in others it was analysed with questions with three options; low, medium and high risk [44]

### Justification For The Study

The available research in this field has been revised and a consensus that included recommendations to carry out epidemiological studies in different areas provided. Given the current situation, studies that inform us of the situation at a level closest to the patient (primary health care) are essential. As are studies that permit us to set out risk levels based on a description of exposition to drugs, riskful behaviour (alcohol intake and consumption of other psychoactive substances) and chronic and acute health conditions, and studies that assess the RTI risk perception of drivers.

On the other hand, the prospective group follow-up can give us an idea of the changes that may appear in terms of RTI risk factors, and in terms of traffic collisions with/without injuries. Without a doubt, this is useful information in the planning of preventative interventions in primary health care.

## Objectives

Main aim: to assess the distribution of the risk factors associated with RTI in a driving population assigned to a group of participating primary health care centres in the city of Barcelona.

Secondary objectives: 1. To study the distribution of RTI risk factors relative to medications, illnesses, alcohol intake and/or consumption of psychoactive substances in the driving population in different groups according to age and gender. 2. To outline the correlation between the number of risk factors related to suffering an RTI and the background of registered collisions, having or not having recieved medical attention. 3. To assess the existing relationship between the self perception level of risk of RTI and the presence of chronic health conditions, medication or consumption of psychoactive substances.

## Methods/Design

### Study design

Cross-sectional and multicentre study.

### Setting

25 primary health care centres in Barcelona.

### Study population

Drivers aged 16 or above who have been attended and who have a clinical register at the participating primary health care centres.

### Sample size calculation

The calculation was carried out using the population of men and women with a driving license in the Barcelona province from the age 16 and over, according to the 2005 census of drivers made available by the General Traffic Department (DGT). For sample calculation purposes, four age groups proportional to women and men were created. Accepting an alpha risk of 0.05 with a precision of +/- 0.05 percentage units in a bilateral contrast for an estimated proportion of 0.5, a random population sample of 385 subjects was determined for each age group. The total sample size is 1540 subjects (Table 3).

**Table 3:** Sample size according to the estimated distribution in age and gender groups

	16-24 yrs	25-34 yrs	35-64 yrs	Above 74 yrs	Total
Men	214	212	228	317	971
Women	171	173	157	68	569

Total	385	385	385	385	1540

### Recruitment Process

**Figure 1:**
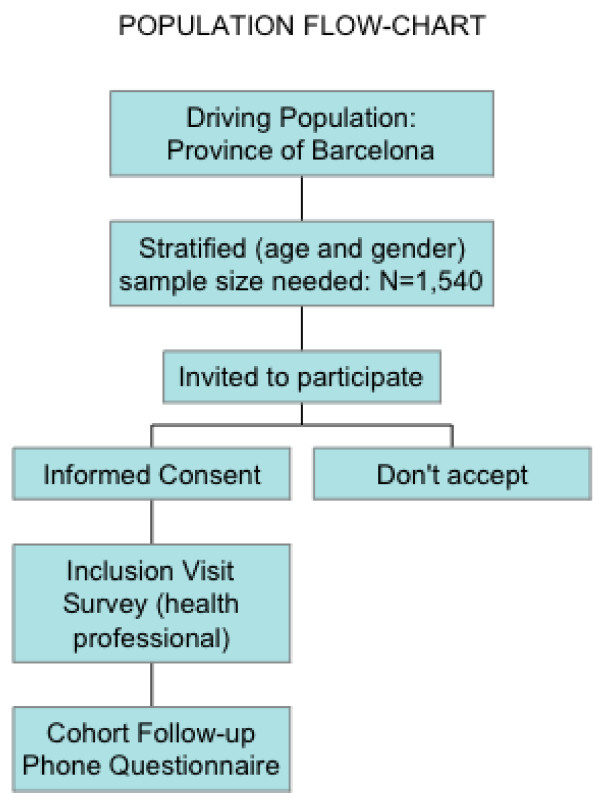
**Flow Chart: Algorithm of the study**.

The study population will be recruited by consecutive sampling. Each collaborating researcher, after verifying the inclusion criteria, will include 16 subjects, two for every age and gender group of those described in the table above. All subjects of 16 years of age or over that consult any collaborating health professionals (nurses or doctors) for any motive, will be invited to participate. They will be informed by the collaborating researcher (patient information leaflet) and asked to take part in the study and in yearly follow-ups via telephone. In case of acceptance, they will read and sign a consent form. Each centre will recruit patients until reaching the number of participants stipulated, taking into account the necessary distribution by age groups and sex. Figure 1 Flow Chart: Algorithm of the study shows the recruitment system and yearly monitoring.

### Selection criteria

#### Inclusion criteria

aged 16 or above; sufficient understanding and knowledge of Catalan or Spanish; possession of valid driving or motorbike license; subject's agreement to participate in the study and in yearly follow ups via telephone through a signed consent form.

#### Exclusion criteria

people who do not poses a telephone contact number; those with serious psychiatric problems; those with physical or psychiatric disabilities that impede their participation in the study; those with a terminal illness.

All centres of Barcelona province and the metropolitan area will be invited to participate, and those who voluntarily accept to participate will be included in the study. *To date, at the end of the recruitment period, 25 practices will participate in the study*.

### Data collection

In the inclusion visit, the health professional will gather the relevant medical data and history (illnesses, medication, alcohol consumption and psychoactive substances) from the patient's clinical record, after checking and completing it together with the patient. Then, relevant data on behaviour and risk-perception of suffering from an RTI, and data on previous traffic accidents will be collected.

A yearly follow up via telephone is expected to be performed.

### Study variables

#### We dispose of two data collection instruments in order to carry out this study

-A survey, which will be carried out by health professionals in a face to face interview. Data will be obtained from the clinical register, and double-checked by the researcher and patient during the visit.

In this survey, the following will be noted: 1. Demographic data: age, gender, civil status, place of origin, level of studies, social class (according to the categories proposed by the Spanish Epidemiology Society). 2. Medication: long-term medication, acute medication (taken in the last 30 days). 3. Chronic health conditions related to higher risk of RTI (Table 3). 4. Alcohol consumption (measured using the AUDIT-C test). 5. Consumption of psychoactive substances (cocaine, ecstasy and others) during the last year, as self-reported by the patient and self-declared dependency level of psychoactive substances in the last month. 6. Estimated level of perception risk, as assessed by the health professional, and rated using a scale from 0 (no risk) to 10 (maximum risk). 7. Driving data: type of driver's license, length of time driver's license held; type of vehicle most usually driven (moped, motorbike, car, others); professional driver (y/n); time spent driving in hours per week; type of road usually used (urban, motorway or others); safety behaviours (use of seatbelts, use of helmet, use of car seats for children and adherence to speed limits). 8. Collision background in the last year (with or without injury); History of medical attention for traffic road injuries in the last year. 9. Self-declared self medicating. 10. Self evaluation of state of health (using the SF-12 test). 11. Self-perception of risk. Individuals will be asked to score their own risk for RTIs, when compared to subjects with their same gender and age, rated in a scale from 0 to 10.

-Telephone survey: In the telephone survey carried out annually by a trained interviewer the following data will be collected and reviewed: 1. Collisions in the last year: date, time of day and day of the week when it occurred; type of road where the collision occurred; vehicle being driven and other vehicles implicated in the crash; medical attention needed (Yes/No/type); need for a sick leave due to traffic injuries (Yes/No/duration); after-effects (Yes/No/type); approximate material costs/vehicle repairs needed (Yes/No/Costs). 2. Newly diagnosed long-term conditions (in the last year). 3. Chronic and acute medication recently taken (time taken and coded according to therapeutic risk group). 4. Self-reported consumption of psychoactive substances. 5. Changes in driving conditions: type of vehicle usually driven, time spent driving in hours per week; type of road usually used (urban, motorway or others); safety behaviour (use of seatbelts, use of helmet, use of car seats for children and adherence to speed limits). 6. Changes in level of self-perception of risk (as reported by the subject of study).

### Statistical analysis

The characteristics of the population will be outlined using a descriptive univariant analysis, calculating the average, standard deviation, median, minimum and maximum values for continuous variables, and frequency and percentages for categorical variables.

The results of the descriptive part will be expressed with their confidence interval (CI 95%). In order to investigate the distribution of the risk factors associated with suffering an RTI according to age and gender, bivariant comparisons will be carried out using the Chi-square test between categorical variables and the Student's t-test between continuous and categorical variables. In order to analyse the association between the risk perception of an RTI (low/medium/high) and the different risk factors considered, an unconditional binary logistical regression model will be implemented. For the risk perception considered as an ordinal scale, a multiple lineal regression will be implemented. For the selection of the variables for the multivariate model, the methodology proposed by Hosmer-Memeshow [57], Cobo [58] and Greenland [59] will be implemented. In the initial multivariate model, the significant bivariate level variables will be introduced, based on the level of significance p = 0.25 and the variables considered to be clinically relevant. Confusion, interaction and co-linearity factors will be analysed. The adjustment of the model will be evaluated using the Hosmer-Lemeshow test and the area under the curve.

All statistical tests will be carried out with a confidence level of 95% and supposing a bilateral contrast. The analyses with be carried out using the statistics package SPSS.

### Quality control

The variables collected during the interview with the health professional will be reviewed and checked against the patient's clinical record during the same visit. Coordination meetings will be held with the collaborating researchers periodically. Any confusing or missing data will be revised by one of the investigators who will ask for explanations from the relevant investigator. Data collection booklets will be read using Teleform technology. The introduction of the data into the database as well as quality control will be carried out by a trained person with ample experience using Teleform. The interviewers carrying out the telephone interview will be trained and accredited periodically in order to ensure the maximum quality of data collection.

### Difficulties and limitations of the study

In this study the relationship of risk factors of suffering an RTI will be evaluated; however, this does not apply to causality assessment, which would need a different specific study design.

There may be subjects who, although are assigned to one of the participating centres, do not have an open clinical register there because it is the first time they have visited the clinic or because the record is on paper. In these cases, the clinical data will be collected during the inclusion visit.

The computerised clinical registers (eCAP™ software or similar) may not be correctly updated in some cases, due to their recent addition to the clinic. The researchers will complete the information in the inclusion visit and through the telephone interview.

There are some difficulties related to the measuring instrument (telephone interview and face to face interview), for ethical and legal reasons (consent form) as well as validity (there are no valid surveys designed to this effect). The researcher will carry out a pilot study to rate the difficulties in completing the survey.

Only those health and medication problems more often found in our field will be analysed in this study.

Alcohol intake and the consumption of psychoactive substances will be considered self reported. Biological tests will not be carried out because the objective is to obtain the data under the same conditions of actual clinical practice carried out in primary care visits.

The generalisation of the results will be limited to an urban population.

### Ethical aspects

The study will be carried out according to the principles contained in the Helsinki Declaration and its successive reviews and the Good Clinical Practice standards. The LESIONAT study protocol obtained approval from the corresponding Ethics Committee (CEIC IDIAP Jordi Gol).

Consent form: the information will be provided verbally and in writing. The study subjects will have sufficient opportunity to ask about study details. Printing of the consent form will follow the rules contained in the Helsinki declaration as stipulated in article 12 under the first title of the Royal Decree 15/1999.

Data confidentiality: in this study, only researchers and monitors/auditors will have access to the information on the participating subjects.

## Discussion

There are various motives that justify the importance of this study. Primary health care is an advantageous location to carry out preventive interventions in RTI. In our country, according to data provided by the Primary Health Care Information System, 75% of the population consults a general practitioner at least in their life, and the average number of visits is between 5 and 6 yearly. Furthermore, public health cover in Spain is practically universal. This makes primary health care an ideal place for the study and implementation of preventive measures in the RTI field. These could include warnings from the side effects of certain medication, the effects of psychoactive substances on driving skills to the importance of the control and monitoring of chronic or acute illnesses that help the patient elaborate a real reception of their physical state and capacity to drive [60-62].

RTI pose health problems that are extremely high in magnitude and in social and health repercussions. The Catalan Public Health Plan contemplates this intervention as a priority objective, and so establishes the following main aims for 2010: 1. To reduce by 20% the mortality rate associated with RTI in the main 3 population groups where they are most frequent (see introduction). 2. To reduce by 50% the mortality rate associated with RTI in men and women from 15 to 24 years old. 3. To reduce by 50% the mortality rates associated with RTI in drivers linked to an excessive intake of alcohol. 4. To reduce by 25% the prevalence of severe injuries associated with RTI in the 3 main population groups (see introduction).

The elements evaluated in this study (medication, chronic health conditions, consumption of psychoactive substances), are often managed in the Primary Care field and are therefore registered in medical records. Moreover, these are aspects that are inherently related to the Primary Health Care field which means that the date is quite complete and difficult to attain in other existing registers.

The improvement in the basic knowledge of risk levels of suffering from an RTI that this study aims to achieve is a necessary first step for the definition of preventative activities in primary health care in the near future.

The research team sets out the possibility of creating a group with the population group, and establishing telephone monitoring within it to investigate the prospective appearance of RTI, and the factors associated with them. With this idea, the included subjects will be asked for their consent, and to provide a contact phone number and a preferred time of day for posterior contact.

Regarding the bibliographic impact of this study and after thoroughly reviewing the available bibliography, we did not find any studies similar to the project that we propose. Therefore, we believe that the originality of the questions that we raise (taken from a primary health care approach) provided added value.

## Competing interests

The authors declare that they have no competing interests.

## Authors' contributions

CM, DP, JB, LR, XM, CP, XB, LG, FM and MB, form the nucleus of the team of researchers in the LESIONAT study research Group. SV, NS and EB took responsibility for the maintenance of the database, as well as processing the manuscript. SV reviewed the long-term conditions section, and NS the long-term drugs references. All of the authors read and approved the final manuscript.

## Note

* "LESIONAT": Niveles de Riesgo de sufrir una **LESION **por **A**ccidente de **T**rafico en atención primaria"

## Pre-publication history

The pre-publication history for this paper can be accessed here:

http://www.biomedcentral.com/1471-2458/10/136/prepub
